# Predicting Road Encounter Hotspots for Infrequently Detected Species Using Opportunistic Data: A Case Study With Blanding's Turtle (*Emydoidea blandingii*)

**DOI:** 10.1002/ece3.73265

**Published:** 2026-03-22

**Authors:** Sean G. Jackson, Alexandra J. Burrows, Glenn Johnson, Eric M. McCluskey, Tom A. Langen

**Affiliations:** ^1^ Institute for a Sustainable Environment, Clarkson University Potsdam New York USA; ^2^ Department of Biology SUNY Potsdam Potsdam New York USA; ^3^ Department of Biology Grand Valley State University Allendale Michigan USA; ^4^ Department of Biology Clarkson University Potsdam New York USA

**Keywords:** hotspot modeling, movement ecology, reptiles, road ecology

## Abstract

For road mitigation measures to prevent roadkill and conserve landscape connectivity to be effective, the measures must be located where animals are most likely to encounter roads. However, accurate identification of road encounter hotspots is difficult when presence records are sparse and collected haphazardly, often the case with small, uncommon species. Blanding's Turtle *Emydoidea blandingii* is a threatened species for which road mortality contributes to population declines. Using opportunistic detections of Blanding's Turtle along roads, we investigated whether it is possible to predict road encounter hotspots throughout an extensive road network with such data. First, we used general linear modeling (GLM) to infer landscape features associated with Blanding's Turtle road encounter records. After locating spatial clusters of encounters, GLM was used to identify landscape features associated with these hotspots. Next, Blanding's Turtle least cost movement paths were delineated within the landscape, and sites where paths crossed roads were located. Blanding's Turtle locations were positively associated with proximity and extent of wetlands, and negatively associated with grasslands and developed land use. Hotspots were located along predicted Blanding's Turtle least cost movement paths, indicating that behavioral movement models are useful for predicting encounter locations. A significant fraction of road encounter records came from a small number of hotspot sites, located along the predicted movement paths. We conclude that it is possible to generate predictive models of road encounter hotspots even when data are sparse, collected opportunistically, and subject to spatial biases in reporting across a road network. These models can be applied throughout a road network to identify road segments that are good candidates for effective road mitigation.

## Graphical abstract

The article focuses on ways of identifying important road encounter locations of a rare species, Blanding's Turtle *Emydoidea blandingii* , using haphazardly collected data, as is usually the case with rare species, in order to appropriately site mitigation measures. We apply three different methods for predicting road encounter hotspots over a large road network and find that each provides helpful information for conservation planning.

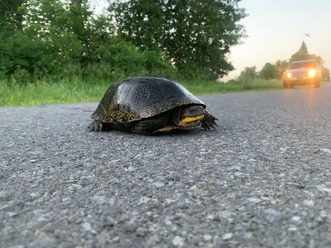

## 1 Introduction

Roadways and road traffic can harm wildlife populations by causing mortality from vehicle collisions, creating barriers to movement across the landscape, degrading habitat adjacent to roadways, and increasing direct disturbance by people (Forman et al. 2003). Mitigation measures designed to reduce harmful road effects on wildlife include cautionary signage for drivers, barriers to road access, passage structures, and roadside habitat management (reviewed in Glista et al. 2009; van der Ree et al. 2015; Andrews, Nanjappa, and Riley 2015; Andrews, Langen, and Struijk 2015). However, since agencies tasked with mitigating road effects on wildlife can only do so on limited segments of roadway because of implementation and maintenance costs, they need to focus activities on locations where the need is greatest (Gunson et al. 2011; Langen et al. 2015; Paemelaere et al. 2023).

There are three principal ways to locate optimal road segments for mitigation (Langen 2009; Gunson and Teixeira 2015; Langen et al. 2012). The first is to collect data on the locations where animals encounter roads, using records of roadkill, detections of live individuals on or near a roadway, or road encounter locations inferred from radiotelemetry or other movement data. Using computer applications that statistically characterize the spatial patterns of road encounters along a road network, one can pinpoint clusters of wildlife road encounters, called hotspots, where mitigation is likely to have the biggest positive benefit (Gunson et al. 2011; Langen et al. 2012; Heigl et al. 2017; but see Zimmermann Teixeira et al. 2017). To identify hotspots within a road network, a high‐quality dataset of georeferenced road encounters is needed for the entire road network of concern. The probability of animal detection and inclusion in the database should be the same across all segments of the road network, or else the spatial patterns, including predicted locations of hotspots, may simply be the misleading consequence of spatial biases in detection and reporting (Jørgensen et al. 2025; Velosa et al. 2025).

A second way to locate optimal locations for mitigation is to identify landscape and road features that are associated with road encounters, ideally in conjunction with data on local and regional distribution of the species of concern (Langen et al. 2009, 2012). For some species, it may be possible to use natural history knowledge about them in conjunction with accurate data on land use and landcover (LULC) dispersion around a road network to locate places that roads cross high‐quality habitat or movement corridors. These are the locations where the likelihood of road encounters is high (Meek 2015).

Alternatively, one can characterize attributes of the road and adjacent LULC at locations where there are records of road encounters. The locations can be compared to either random locations in the road network (also referred to as “background points”), or else comparison locations where there are no road encounter records of the species, referred to as “pseudoabsences.” Using exploratory general linear modeling (GLM) statistical techniques, one can identify landscape and road features that are associated with wildlife road encounters generally or hotspots specifically. With aid of a geographic information system (GIS), one can apply a model using these predictors to locate other places in the road network that match these attributes, and hence are candidates for mitigation (Langen et al. 2009, 2012; Blackburn et al. 2021). The key to accurately identifying landscape and road features associated with road encounters is to have comparison points (background or pseudoabsences) that are appropriately matched to the road encounter records, within the same landscape and along the same roads as the encounter data. Doing so minimizes the risk that spatial biases in sampling effort or inclusion of areas where the species is not present result in misleading or invalid road encounter predictors.

The third approach to locating road segments for mitigation is to predict where animals encounter roads by modeling how they would likely move through a landscape (Cushman et al. 2014; Fullman et al. 2021; Aiello et al. 2023). Animals' movement trajectories can be predicted if data on LULC, topography, hydrology, and other landscape features affecting animal movement are available in a GIS at the appropriate spatial scale, and if it is known what features are preferred or avoided when an individual animal moves through a landscape. The latter implies that the natural history of a species must be well understood, especially its habitat preferences. Using modeled movement trajectories, places where animals are predicted to encounter roads when following preferred pathways are candidates for mitigation. A movement model may provide a powerful way to identify candidate locations for mitigation, even in the absence of road encounter data. However, it is essential that the model be adequately validated first with data on actual road encounters before much confidence is placed in a movement model's predictions (Iverson et al. 2024).

Freshwater turtles are highly vulnerable to road disturbance, including roadkill and road barriers to movement, because of their demography, movement behavior, slow speed, and small size (Andrews, Nanjappa, and Riley 2015; Andrews, Langen, and Struijk 2015; Jackson et al. 2015a; Langen and Colino‐Rabanal 2026). Mitigation measures for turtles include signage that alerts drivers to be cautious, fencing or walls that prevent road access, modified box or pipe culverts under roads for safely traversing the roadway, and constructed nesting habitat to entice female turtles to nest away from roadsides (Jackson et al. 2015b; Crawford et al. 2017; Boyle et al. 2021; Langen and Colino‐Rabanal 2026). Resources are limited for mitigation, however. To be effective, turtle mitigation efforts must be targeted at the most impactful locations.

The Blanding's Turtle ( *Emydoidea blandingii* ) is a semiaquatic turtle species of temperate northeastern and north‐central North America that is listed as a species of conservation concern in most US states and Canadian provinces where it occurs (King et al. 2021; Auge et al. 2024). This turtle is classified as threatened in New York State, and roadkill is listed as a major threat to populations (Ross and Johnson 2018), as it is elsewhere within the species range (King et al. 2025). A major stronghold of Blanding's Turtle in New York is in the St. Lawrence River Valley (SLRV), and this species has been subject to studies on distribution, habitat associations, and genetic structure (McCluskey et al. 2016, 2022; Stryszowska et al. 2016; Jordan et al. 2024).

Blanding's Turtles encounter roads when moving overland to access waterbodies or nesting sites, and do not avoid crossing roads that they encounter (Beaudry et al. 2008). Blanding's Turtle roadkill consists predominantly of adult females, and occurs during nesting movements (Steen et al. 2006; Beaudry et al. 2010). There have been several attempts to predict where Blanding's Turtles encounter roads, by predicting Blanding's Turtle movement patterns (Beaudry et al. 2008; Litvaitis and Tash 2008; Mui et al. 2017); these studies were not validated with road‐occurrence data.

The population density of Blanding's Turtle is low in the SLRV, relative to the most prevalent turtle species (Painted Turtle *Chryemys picta* and Common Snapping Turtle *Chelydra serpentina* ), and turtles of this species are rarely encountered during structured road searches—too few to do conventional spatial hotspot analyses (e.g., 2 Blanding's Turtles in a 6564 road‐km survey, Langen et al. 2012). Indeed, most Blanding's Turtle road‐encounter studies focus on documenting patterns of mortality at known hotspots (e.g., Markle et al. 2017). However, Blanding's Turtles are occasionally encountered serendipitously along roads. For over two decades, we aggregated records of turtles that we, or members of the public, encountered by chance along roads. Such opportunistic data are subject to severe spatial biases, being predominately from the subset of roads most frequently driven by people who recognize the species, know how to report road encounters, and are willing to do so.

In this study, we attempted to identify candidate locations for road mitigation to benefit Blanding's Turtle throughout the SLRV road network, using multiple methods. First, we used a GLM approach to infer landscape features associated with the Blanding's Turtle road encounters. Second, we located spatial clusters of road encounters, and again statistically identified landscape features associated with these hotspots. Third, we predicted Blanding's Turtle movement patterns within the landscape, and located areas that movement paths transected roadways. Finally, we compared the predicted hotspots based on the modeled movement trajectories with the actual locations of Blanding's Turtle road encounter hotspots. Our goal was to answer the question: *Is it possible to predict road encounter hotspots throughout an extensive road network for an infrequently detected species using sparse, haphazardly collected data?*

## 2 Materials and Methods

### 2.1 Study Area and Data Source

**FIGURE 1:**
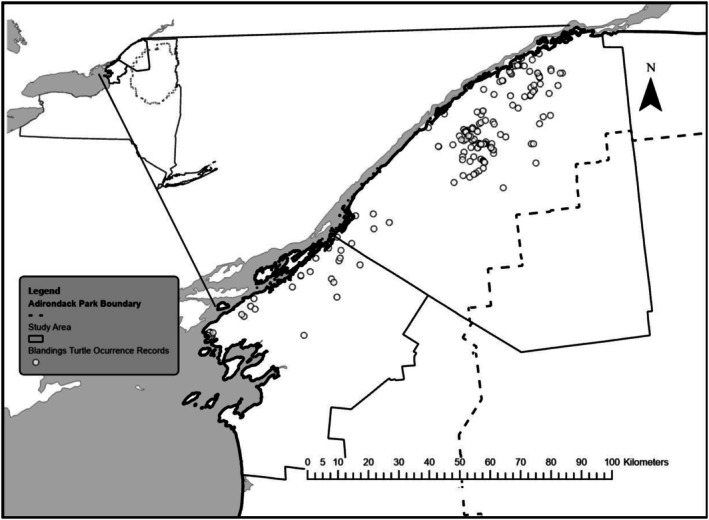
Study area encompassing the SLRV region of New York State containing Blanding's Turtle road encounter records.

The study area encompasses lowland sections of Saint Lawrence and Jefferson counties in the SLRV of northeastern New York, USA, an expanse of 8000 km^2^ crossed by 11,271 km of roadway (Figure 1). This is the same region as was the focus of studies on Blanding's Turtle distribution, landscape permeability, and genetic structure in Stryszowska et al. (2016) and McCluskey et al. (2022). The region has strong seasonality with long, cold winters and short, mild summers, resulting in a 125‐day growing season; turtles are active on land from early May to early October. LULC in the SLRV is largely composed of deciduous or mixed deciduous‐evergreen forest, hayfield or pasture, maize cultivation, and wetlands (Table 1). The area is rural, indicated by the low proportion of developed land use and high proportion of agricultural land use and natural landcover. Population density is low, about 22 people/km^2^.

**TABLE 1:** Land cover/use composition of the study region in northeastern New York, as classified by the 2019 National Land Cover Database.

NLCD classification	Percent coverage
Deciduous Forest	37.40
Hay/Pasture	16.40
Woody Wetlands	15.99
Evergreen Forest	7.75
Cultivated Crops	5.37
Mixed Forest	3.65
Developed, Open Space	3.01
Open Water	2.53
Shrub/Scrub	2.13
Emergent Herbaceous Wetlands	1.67
Developed, Low Intensity	1.49
Herbaceous	1.36
Developed, Medium Intensity	0.85
Developed, High Intensity	0.25
Barren Land	0.16

Blanding's Turtle road occurrence records were collected by coauthor GJ between 1997 and 2021; nearly all records were collected after 2015. Records included data from structured road surveys for turtles, opportunistic encounters while traveling to fieldwork sites or other activities by the authors and their students, and carefully vetted reports from the public. The records include roadkill and live individuals on the roadway or at the roadside. For reports from members of the public, we contacted the individual and requested an image of the animal or its remains, and went to the location with them to collect an accurate georeference. Records were classified as a valid road occurrence if the location point was a georeference within 10 m of the roadway within the study region. This distance was used to account for errors in georeferencing information and to include road verge occurrences of turtles nesting alongside the road or preparing to cross it. Because of small differences in the geographic extent of included turtle records in different analyses described below, there is a small difference in the number of included Blanding's Turtle records depending on the analysis. A total of 249 Blanding's Turtle road records were included in this study, including 145 mature female turtles (58% of records) and 28 mature males (11%); the remaining were juveniles or unsexed adults (30%). The female bias in the road encounter records is the result of nesting movements (Steen et al. 2006). Most records were in June, which is the peak time of nesting in the region, and 43% of females in the dataset were recorded as gravid. In addition to the road occurrence database, we had a database of Blanding's Turtle occurrence locations within the region based on wetlands trapping surveys, including 54 sites with Blanding's Turtle captures and 64 sites with no Blanding's Turtle detected (see McCluskey et al. 2022).

### 2.2 Spatial Reference and Data Sources

We performed all geospatial analysis using either QGIS 3.2 (https://qgis.org/) or ArcGIS Pro 3.1 (https://www.esri.com/en‐us/arcgis/products/arcgis‐pro/overview). The spatial reference used for all geospatial analyses was NAD 1983 UTM Zone 18 N, which maintains geometric and positional accuracy for New York State. Imported geospatial data used for this analysis included the 2019 National Land Cover Dataset (NLCD, https://www.mrlc.gov), New York State Simplified Streets geodatabase (https://data.gis.ny.gov/), and New York State Municipal and County Boundaries (https://data.gis.ny.gov/). We acquired National Agriculture Imagery Program orthoimagery (0.093 square meter spatial resolution) from the Geospatial Data Gateway (https://datagateway.nrcs.usda.gov/), and New York State wetland inventory polygons from the New York State GIS Clearinghouse (gis.ny.gov). All rasters used for our analyses were 30‐m resolution, because this is the raster size of the National Land Cover Dataset. We derived all subsequent layers generated during this analysis from these data and the georeferenced occurrence data using the ArcGIS suite, KDE+ toolbox for ArcGIS (https://www.kdeplus.cz/en/), and the Linkage Mapper toolbox for ArcGIS (https://linkagemapper.org/). All geoprocessing and data preparation were performed on a desktop computer with 8 GB of RAM and a 2.1 GHZ processor.

### 2.3 Landscape Predictors of Blanding's Turtle Road Encounter Locations

For each Blanding's Turtle road encounter record, a matching null point (= pseudoabsence) was assigned along the same road at 1.0 km road distance from the turtle record in either direction along the same roadway. Thus, null points were on the same roadways and in the same landscape as the turtle road encounters. Our justification for this 1.0 km pairing was that the probability a turtle was detected and reported to us was likely the same along a segment of the same road, and so systematic differences in attributes between turtle record locations and nearby locations on that road are likely to be informative. Preliminary examination of landscape features indicated that spatial autocorrelation of LULC declined to background at under 1.0 km between points, again making 1.0 km a suitable distance for a paired comparison point.

Only null points 1.0 km from any turtle encounter record were retained; 149 null points were included in the final analysis. The number of null points is lower than the number of turtle records because some turtle records were located such that a 1.0 km putative null point was less than 1.0 km from another turtle record; in such cases, we used the nearest null point that was greater than or equal to 1.0 km for any turtle record.

At each point (encounter or null), we measured the straight‐line distance to the nearest wetland (as indicated by the orthoimagery and wetland polygons) on each side of the road and the minimum straight‐line distance between the two wetlands passing through the point using a GIS measuring tool. Three buffers (50, 100, 250 m) were placed around each point to characterize LULC as indicated by the NLCD. Three buffer sizes were used because we did not know a priori what scale was most relevant for characterizing the local landscape affecting Blanding's Turtle movement. Using the ArcGIS zonal statistics tool, we computed the pixel coverage of each LULC category within each point's buffer, for each of the three buffer sizes. The NLCD LULC categories were then consolidated into five LULC categories: (1) *open water*; (2) *developed land*, including developed open space and low, medium, and high intensity development; (3) *forest*, including deciduous, evergreen, and mixed forests; (4) *grassland*, including pasture/hay, cultivated crops, grassland, and scrub/shrub; and (5) *wetlands*, including woody and emergent herbaceous wetlands.

We compared LULC variables (three wetland distance and five landcover variables) of Blanding's Turtle records to null points at the three spatial scales in two ways. First, we compared mean values of each LULC variable of all Blanding's Turtle records (*N* = 249) to null points (*N* = 149). Second, we paired a turtle record with its matched null point (point 1.0 km on the same roadway) and compared the difference in each LULC variable using a paired *t*‐test (*N* = 149 matched pairs); we confirmed normal distributions of the differences between pairs. The effect size was calculated using Cohen's D, where a small effect is conventionally categorized, using the absolute value, 0.2 ≥ D ≤ 0.5, and medium effect size is 0.5 ≥ D ≤ 0.8 (Sullivan and Feinn 2012); we had no large effect sizes.

The six LULC categories that were indicated to be predictive in univariate analyses, based on statistical significance, were used in an exploratory multiple logistic regression (logit link, binomial error distribution) including wetlands, developed land, grassland, and the three wetland distance measures. Note that two predictors (*Distance to the Nearest Wetland*, *Distance to the Opposite Wetland*) were each correlated with *Distance Between the Nearest Opposing Wetlands*; this should not be a problem in this analysis because model overfitting by adding two redundant predictors is penalized for the additional parameter. We used the 100 m buffer LULC data, given that significant predictors had the highest effect sizes at this scale in the univariate analyses. Using the *dredge* function in R Studio (https://cran.rstudio.com/), we evaluated all combinations of the LULC variables, including the saturated model (all six predictors) and the intercept model (no predictor); a total of 64 models were evaluated. Models were ranked by their Akaike Information Criterion (AIC) value, with the lowest AIC indicating the best fit. Following convention, we considered models within 2.0 AIC units of the best fit model to be equally supported. The accuracy of the best model at discriminating turtle points from random points was evaluated using Cohen's kappa, a statistic that was developed for inter‐rater agreement but also used for comparing dichotomously classified model predictions with data, especially when the two classes have unequal frequencies; the statistic is conventionally interpreted as *k* = 0.01–0.20 as slight agreement, 0.21–0.40 as fair agreement, 0.41–0.60 as moderate agreement (McHugh 2012).

### 2.4 Locating Road Encounter Hotspots

We imported a road network geodatabase containing all major roads in New York State into ArcGIS Pro and clipped to the study area. Most roadways in the study area road network had no records of Blanding's Turtle road encounters. While the absence of records could be due to the true absence of road encounters, it may also be due to less frequent sampling than road segments with records. To avoid the biases associated with treating no‐data roads as true absences, using a larger database of occurrence records that also included occupancy records in wetland habitat nearby the roadways, road segments that did not have any Blanding's Turtle occurrences within 100 m somewhere along them were removed from the road network. The remaining road network was then converted to *polyline‐z* so that it could be linearly referenced. The *create route* and *find along routes* functions resulted in a table with each line segment and the position of a Blanding's Turtle occurrence along each segment. Our choice to exclude roads with no turtle records along or nearby them addressed detection biases, but we were cognizant that this was at a cost of potentially limiting generalizability outside the included road network, at least until further model validation was done.

KDE+ is a data analytical tool that identifies hotspots of road encounters along linear networks using kernel density estimation (Bíl et al. 2013; https://www.kdeplus.cz/en/). It can be applied using spatial data in ArcGIS or using tabular data in its standalone application. Both methods were applied in this study. Occurrence records that were within 10 m of a road segment were tethered to that road segment and their distance from the origin of the road segment recorded. For the ArcGIS toolbox, the shapefiles containing point data (Blanding's Turtle occurrences) and selected roads in the network were used to generate 100‐m and 200‐m bandwidth hotspots, based on our prior research documenting the turtle roadkill hotspots are around those lengths in our region (Langen et al. 2012), and no a priori reason to select one bandwidth over the other. The output was two shapefiles displaying the location and associated attribute tables for the 100‐m and 200‐m hotspots. There was little qualitative difference in results with the two bandwidths. The linear referenced tabular format was entered into the KDE+ stand‐alone application for a 200 m bandwidth. Hotspots were ranked using cluster strength, a ranked numerical score of the density of records relative to background, interpreted as the intensity of a hotspot (Bíl et al. 2013).

### 2.5 LULC Predictors of Road Occurrence Hotspots

We delineated 250 m buffers around each hotspot centroid from the KDE+ analysis and each occurrence record. In addition, 1697 null (= nonhotspot) points located greater than 250 m from a hotspot centroid were generated along the trimmed road network, and a 250 m buffer delineated around each. We selected a 250 m buffer based on prior work that indicated that this radius struck a balance of not being too granular nor too coarse; other spatial scales (100, 500 m) provided qualitatively similar results. Note that buffers could overlap, especially for clustered turtle occurrence points at hotspots, resulting necessarily in spatial nonindependence in points located less than 250 m apart. The *tabulate areas* function was performed using the LULC within each buffer, yielding a tabular representation of landscape composition. Field calculations were performed in the attribute table to convert the raw area to a composition percentage for each land cover category. Treating each buffer as an independent replicate, using nonparametric Mann–Whitney tests, we statistically compared the median percent LULC class at hotspots to the null points. A nonparametrical statistical test was used since tests for normality using a Shapiro–Wilk test indicated significant deviations from it due to positively skewed data.

### 2.6 Landscape Connectivity Analysis

We performed concurrent least cost pathway (LCP) analyses using the Linkage Mapper ArcGIS toolbox. To run Linkage Mapper, core habitat nodes are connected by calculating least cost pathways (McRae and Kavanagh 2011). Wetlands were treated as core habitat nodes in our study. The NLCD has several wetland categories. To reduce the number of polygons generated in the study area, all of these wetland classifications were reclassified into a new raster with all wetlands sharing a uniform value. The *raster to polygon* function was used to convert the wetland raster layer into individual polygons. Polygons were considered core habitat nodes if they met both of the following criteria: (1) the wetland polygon contained a Blanding's Turtle record, and (2) the polygon was greater than 10,000 m^2^ (1 ha). This wetland area threshold was used to keep the analysis within the computational capacity of the desktop computer. Wetlands exceeding the area threshold are much more likely to provide occupied habitat than smaller ones, based on our occupancy surveys (Stryszowska et al. 2016). These criteria reduced the total number of wetlands polygons from 114,700 polygons to 117. These polygons were compared to high‐resolution orthoimagery to validate their accuracy. Admittedly, by excluding smaller wetlands, we risked failing to detect movement paths between such features, which Blanding's Turtles are certainly known to use (Beaudry et al. 2009).

**FIGURE 2:**
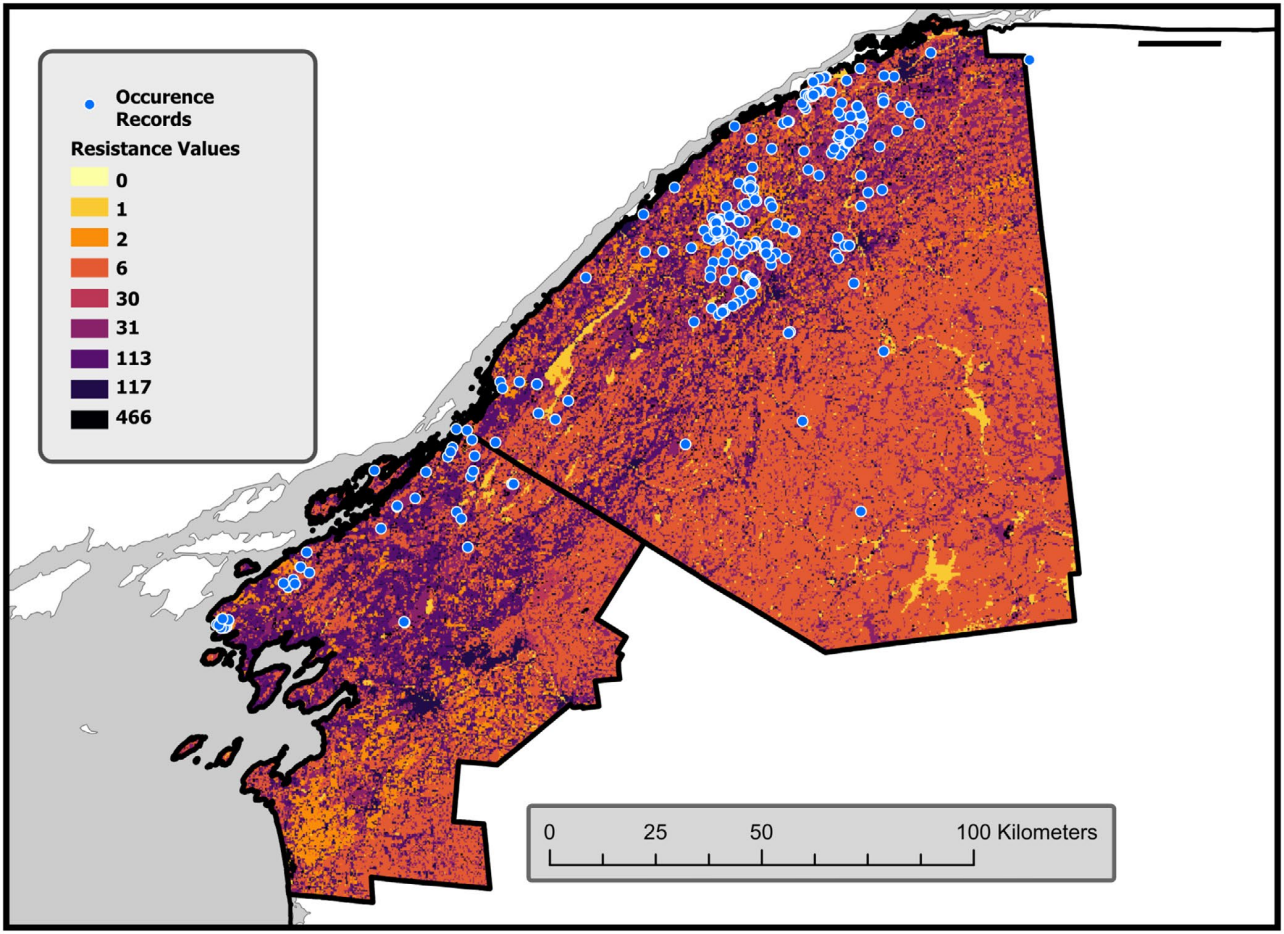
Resistance surface after reclassifying NLCD 2019 to genetic connectivity resistance values from McCluskey et al. (2022). Lighter colors (lower resistance values) indicate putative preferred LULC for movement paths.

A resistance surface is a raster layer where each raster cell is assigned a numeric value that defines the ease in which a target species moves across features of the landscape. The resistance values may be derived from expert inference but more preferably from movement or genetic data (Zeller et al. 2012; Milanesi et al. 2017). We adopted the genetic connectivity resistance values used in McCluskey et al. (2022). These resistance values were developed specifically for the Blanding's Turtle population in our region. The resistance surface used for our study was generated by using the reclassify function in ArcGIS Pro 3.1 to reclassify NLCD and then apply the resistance values, rounded to the nearest integer (Table 2). *Resistance GA* (Peterman 2018) was used to create the resistance surface (Figure 2).

**TABLE 2:** Raster reassignment displaying original and reclassified resistance values used to build least‐cost pathways (LCPs).

NLCD classification	NLCD value	Resistance value
NODATA	0	0
Open Water	11	1
Developed Open Space	21	117
Developed, Low Intensity	22	117
Developed, Moderate Intensity	23	117
Developed, High Intensity	24	117
Barren Land	31	113
Deciduous Forest	41	6
Evergreen Forest	42	6
Mixed Forest	43	6
Shrub/Scrub	52	6
Herbaceous	71	30
Hay/Pasture	81	113
Cultivated Crops	82	2
Woody Wetlands	90	30
Emergent Herbaceous Wetlands	95	466

We used the ArcGIS linkage mapper toolbox to examine habitat connectivity across the study area. Due to the size of the study region and subsequent scale of the dataset, the study region was partitioned into 13 subregions using the *split raster* function, because for a conventional desktop computer the computational demands of an unpartitioned dataset were intractable. Additionally, the partitioning allowed us to omit five subregions of the study area that lacked Blanding's Turtle occurrence records, leaving eight subregions for analysis. The inputs for this toolbox included the resistance surface and the wetland habitat node polygons. Attempts to run Linkage Mapper were made for each of the selected subregions until all cost‐weighted distance rasters and LCPs were generated. Run times for Linkage Mapper in each subregion ranged from 35 min to 2 h based on the area and total number of nodes within the subregion.

It is important to note that roadways were ignored when creating the resistance surface, except to the extent that roadside verges are indicated as “developed open space” in the NLCD. We treated roads as having no impact on deterring turtle movement, that is, Blanding's Turtle are “non‐responders” sensu Jacobson et al. (2016), as indicated in Beaudry et al. (2008). In other words, we assumed that Blanding's Turtle encounter roads along movement paths determined by the overall landscape resistance, regardless of the presence of the roadway.

### 2.7 Comparing Hotspots to Predicted LCP

We compared the distances of hotspots versus null points to LCPs. Using the *near* tool, LCPs were tethered to their nearest respective point or line of interest. The *near* tool was applied to all 200 m bandwidth hotspots and null points. Hotspot distances versus null point distances to LCPs were statistically compared using a Mann–Whitney test since preliminary analysis indicated the data distributions were not normal due to positively skewed data.

## 3 Results

### 3.1 Landscape Predictors of Blanding's Turtle Road Encounter Locations

#### 3.1.1 Univariate Analysis of All Points

Turtle road encounters were significantly closer to wetlands on both sides of the road than null points (Table 3); they were a mean distance of 124 m from the nearest wetland, averaging 86 m closer than null points, and the distance between wetlands at turtle points averaged 345 m, 218 m closer than null points. At each of the three spatial scales, turtle road encounter locations had significantly more wetland, lower developed land, and lower grassland coverage than null points. Effect sizes were highest for grassland and wetland at the 100 m scale and highest for developed land at the 250 m scale (Table 3). Judged at the scale with the highest effect size, Blanding's Turtle points were 23% grassland, 28% wetland, and 9% developed; these points had 20% less grassland, 5% less developed land, and 20% more wetland than null points.

**TABLE 3:** Summary table of all Blanding's Turtle road encounter points (*N =* 249) versus null points (*N =* 149) at three spatial scales. Significant *p* values (*p* < 0.05) are in bold. Distances are in meters, LULC classes are proportions.

		Turtle		Null				
		Mean	Standard Deviation	Mean	Standard Deviation	*t*‐stat	*p* value	Cohen's D
	Nearest Wetland	123.5	129.4	209.1	153.7	−5.6	**< 0.001**	−0.62
	Opposite Wetland	252.9	229.1	407.0	247.5	−6.0	**< 0.001**	−0.65
	Distance Between	344.9	308.4	562.4	347.7	−6.1	**< 0.001**	−0.67
50 m	Open Water	0.01	0.06	0.01	0.05	0.5	0.6	0.05
	Developed	0.42	0.21	0.48	0.15	−3.1	**0.002**	−0.31
	Forest	0.15	0.23	0.14	0.20	0.6	0.5	0.05
	Grassland	0.17	0.23	0.32	0.23	−6.6	**< 0.001**	−0.68
	Wetland	0.21	0.27	0.05	0.16	6.9	**< 0.001**	0.65
100 m	Open Water	0.02	0.10	0.01	0.06	1.0	0.3	0.10
	Developed	0.22	0.12	0.28	0.12	−4.8	**< 0.001**	−0.49
	Forest	0.20	0.26	0.21	0.24	0.1	0.9	−0.01
	Grassland	0.23	0.28	0.43	0.28	−7.1	**< 0.001**	−0.73
	Wetland	0.28	0.31	0.08	0.17	8.2	**< 0.001**	0.74
250 m	Open Water	0.02	0.08	0.02	0.08	−0.3	0.8	−0.02
	Developed	0.09	0.06	0.14	0.11	−5.3	**< 0.001**	−0.63
	Forest	0.25	0.25	0.26	0.23	−0.5	0.6	−0.07
	Grassland	0.27	0.28	0.45	0.30	−6.2	**< 0.001**	−0.64
	Wetland	0.30	0.30	0.13	0.17	7.0	**< 0.001**	0.65

#### 3.1.2 Univariate Analysis of Paired Points

Using the 149 matched pairs of Blanding's Turtle road encounter and null points located 1.0 km apart on the same roadway, turtle points were significantly closer to wetlands on both sides of the road than null points; on average 77 m closer to the nearest wetland, 114 m closer to the nearest wetlands on the opposite roadside, and opposing roadside wetlands 170 m closer than null points. At each of the three spatial scales, Blanding's Turtle points had greater wetland coverage (around 10% more) and lower grassland coverage (10% lower) than null points (Table 4).

**TABLE 4:** Summary of paired *t*‐tests between matched Blanding's Turtle and null points (*N =* 149) at three spatial scales. Significant values (*p* < 0.05) are in bold. Distances are in meters. LULC classes are proportions. Means and standard deviations are of the differences between the matched turtle and null point pairs.

		Mean	Std Dev	*t*‐stat	*p*
	Nearest wetland	−77.2	179.5	−5.3	**< 0.001**
	Opposite wetland	−113.9	303.4	−4.6	**< 0.001**
	Distance between wetlands	−169.6	408.6	−5.1	**< 0.001**
50 m	Open water	0.01	0.09	0.8	0.4
	Developed	−0.00	0.19	−0.1	0.9
	Forest	−0.01	0.25	−0.4	0.7
	Grassland	−0.09	0.30	−3.6	**< 0.001**
	Wetland	0.09	0.23	4.6	**< 0.001**
100 m	Open water	0.01	0.10	1.1	0.3
	Developed	−0.01	0.14	−0.8	0.4
	Forest	−0.01	0.30	−0.4	0.7
	Grassland	−0.11	0.36	−3.6	**< 0.001**
	Wetland	0.11	0.27	5.0	**< 0.001**
250 m	Open water	0.00	0.10	0.1	0.9
	Developed	−0.02	0.11	−2.0	0.05
	Forest	0.01	0.26	0.5	0.6
	Grassland	−0.09	0.32	−3.4	**0.001**
	Wetland	0.09	0.23	4.7	**< 0.001**

#### 3.1.3 GLM Using All Points

A multiple logistic regression model comparison indicated that some measure of wetland distance and proportional coverage of developed land, grassland, and wetlands provided the best fit model, as indicated by AIC (Table 5); the top model was better than the saturated model (all predictor variables included, delta AIC = 3.5) and the intercept model (no predictor variables included, delta AIC = 104.5). Like the univariate analyses, Blanding's Turtle points were closer to wetlands, had less grassland and developed land, and had more wetland coverage within the 100 m buffer. The best fit model was:

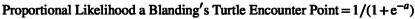

where *a* = 2.06 −0.001*(Distance Between Nearest Wetlands) −3.2*(Proportion of 100 m Buffer Developed Land) −1.5*(Proportion of 100 m Buffer Grassland) + 1.7*(Proportion of 100 m Buffer Wetland).

**TABLE 5:** Summary table of multiple logistic regression models, ranked from the lowest AIC. Included are the top six models, the saturated (all predictor variables) model, and the null model (no predictors, intercept only model).

Model	AIC	Delta AIC	AIC Weight
Btw, Dev, Grass, Wet	448.6	0	0.28
Opp, Dev, Grass, Wet	449.3	0.7	0.20
Near, Opp, Dev, Grass, Wet	450.1	1.5	0.13
Opp, Btw, Dev, Grass, Wet	450.5	1.9	0.11
Near, Btw, Dev, Grass, Wet	450.5	1.9	0.11
Near, Dev, Grass, Wet	451.3	2.7	0.07
Full: Near, Opp, Btw, Dev, Grass, Wet	452.1	3.5	0.05
Null	553.1	104.5	0.00

Abbreviations: Btw, Distances between nearest opposing wetlands; Dev, Land classified as developed; Grass, Land classified as grassland; Near, Distance to nearest wetland; Null, Intercept model; Opp, Distance to opposite wetland; Wet, Land classified as wetlands.

Among points that the model indicated had a 75% or greater probability of being a turtle road encounter point (*N* = 136), 88% were classified correctly. Among points that the model indicated had a 25% or lower probability of being a Blanding's Turtle point (*N =* 15), 87% were classified correctly. Cohen's Kappa = 0.25, which is conventionally interpreted as fair agreement between observed and predicted.

### 3.2 LULC Composition Around Road Occurrence Hotspots

A total of 24 hotspots were located by KDE+, with a mean ± SD road length of 231 ± 117.3 m (median = 235 m, range 35–521 m). Hotspots comprised a cumulative 5.5 km of 264.0 km (= 2%) of roadways that had at least one Blanding's Turtle record. A total of 41% of all Blanding's Turtle road encounter records occurred at these hotspots, and 6% of road encounters were recorded at one 274 m stretch of roadway with the highest cluster strength.

Hotspots had significantly less low intensity developed land cover within 250 m than null (nonhotspot) points, and a similar trend for medium and high intensity development (Table 6). Combining the three development classes, null points had 2.6 times more developed land within 250 m than hotspots (4% hotspot vs. 10% nonhotspot). Developed open space did not differ between hotspots and null points, likely because this land use class includes the mowed roadside verge, which is similar in extent along all local roadways. Woody wetlands had 2.4 times greater coverage within the hotspot centroids than null points (42% hotspot vs. 18% null). Emergent herbaceous wetlands were similar but not as strong in magnitude (3% hotspot vs. 2% null). Nonhotspot points had 1.9 times greater grassland cover than hotspot buffers (14% hotspot vs. 26% null). No other LULC classes differed between hotspot and null locations.

**TABLE 6:** Differences in land cover composition in 250 m buffer zones surrounding hotspots and null points along the road network. Significant values (*p* ≤ 0.05) are in bold.

NLCD land cover class	Mean % null	Mean % hotspot	SD null	SD hotspot	*p*	Mann–Whitney *U*
Open Water	1.28	1.36	4.85	3.91	0.7	0.5
Developed Open Space	9.09	8.33	4.78	3.91	0.4	0.2
Developed Low Intensity	6.33	2.73	6.10	2.87	**0.001**	3.0
Developed Medium Intensity	3.06	0.94	5.30	1.09	0.08	1.4
Developed High Intensity	0.43	0.06	1.15	0.16	**0.04**	1.7
Deciduous Forest	18.78	17.05	16.20	20.53	0.1	1.2
Evergreen Forest	3.61	1.93	8.74	4.30	0.08	1.4
Mixed Forest	2.74	2.52	4.19	3.62	0.6	0.2
Shrub Scrub	1.03	1.55	2.98	4.67	0.5	0.1
Herbaceous	1.06	0.71	2.20	1.95	0.09	1.3
Grassland	25.63	13.46	21.80	18.71	**< 0.001**	3.2
Cultivated Crops	5.95	4.58	12.62	9.56	0.5	1.0
Woody Wetlands	17.73	42.20	19.06	24.62	**< 0.001**	5.0
Emergent Wetlands	2.16	2.81	4.80	3.83	**0.02**	2.1
Low, Med, High Developed	9.83	3.72	11.25	3.17	**0.003**	2.7

### 3.3 Hotspot Distances From the LCPs

**FIGURE 3:**
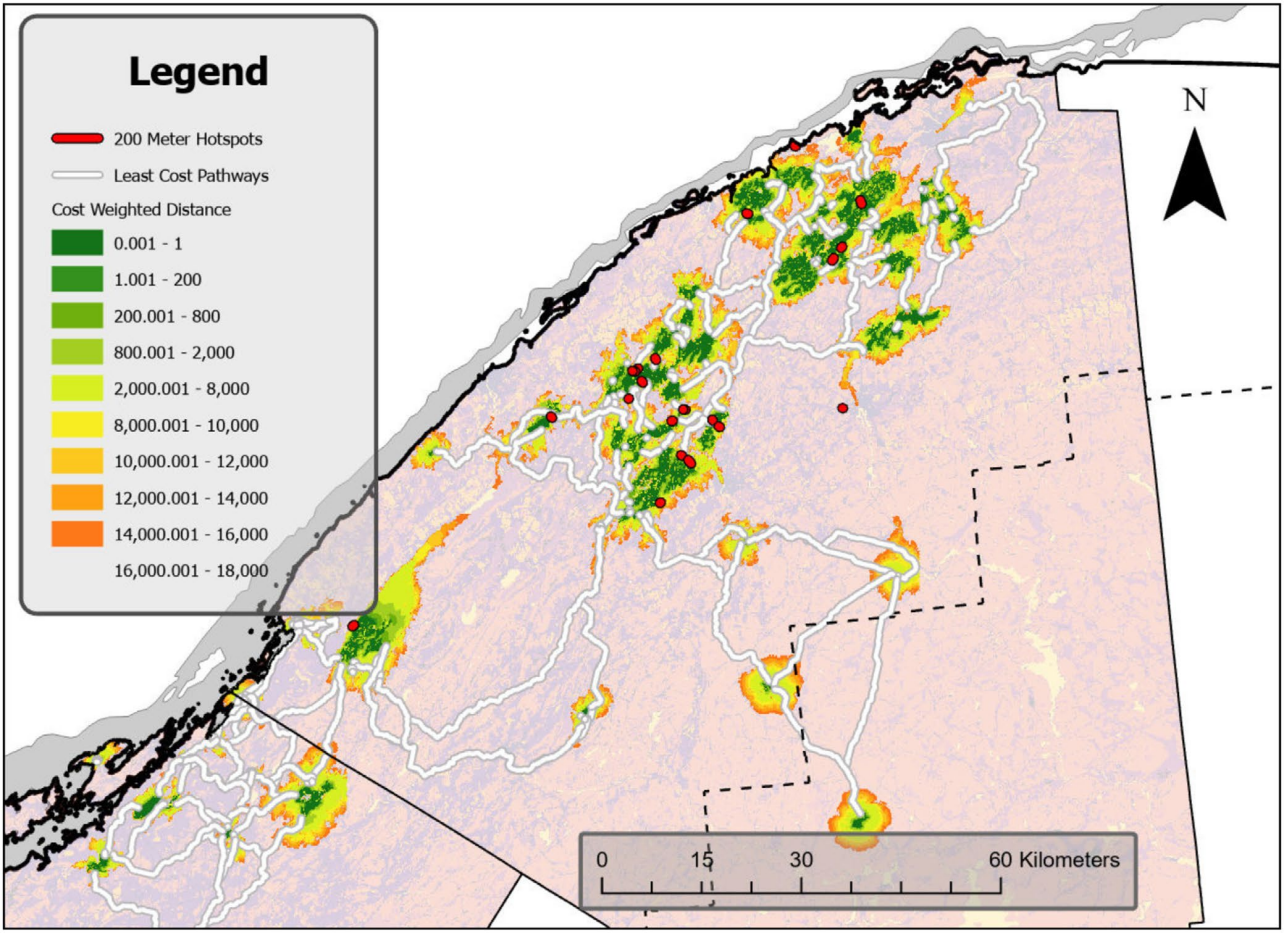
Visual representation of the linkage mapper outputs displaying LCPs, hotspots, and occurrence records overlaid by the cost‐weighted distance raster.

One‐third of hotspots (8 of 24) coincided with an LCP (Figure 3). Null points were 2.8 times farther from an LCP (median distance = 589 m, range: 0.1–7518 m) than a hotspot (209 m, 0–6003 m, Mann–Whitney *U* = 3.1, *p* < 0.001).

Two hotspots were much farther from an LCP than the remaining hotspots (3114 and 6003 m, other 22 hotspots median = 139 m, range 0–1739 m). Additionally, these two hotspots were isolated from other clusters of road encounters: One was located on an island in the northern edge of the study area, and the other near the village of Potsdam, NY, which is much more developed than most of the study area.

One potential bias in our analysis was that hotspots are segments with a length, whereas null locations are a point; the spatial extent of hotspots could result in them being closer to LCPs for this geometric reason. To correct for this, we reanalyzed after adding 116 m distance to hotspots, half the mean hotspot length or equivalent to the hotspot centroid. The results were qualitatively unchanged (Mann–Whitney *U* = 1.7, *p* = 0.04). There was no relationship between hotspot length and distance to an LCP (Spearman rank correlation = −0.14, *p* = 0.5; results qualitatively the same with the two outliers excluded).

Though road segments crossed by LCPs comprised only 25% of the length of the reduced road network (roadways with at least one turtle road occurrence record on or nearby = 264.0 km), 41.5% of Blanding's Turtle road occurrence records were located within LCPs.

## 4 Discussion

We conclude that Blanding's Turtle road encounter locations are predictable, at fine spatial scales relevant for road mitigation, using widely available data on LULC and wetland dispersion within a landscape. Blanding's Turtle encounters are positively associated with proximity and spatial extent of wetlands, especially woody wetlands, and are negatively associated with proximity and extent of grasslands and developed land use. Observed turtle road encounter hotspots coincide with predicted Blanding's Turtle LCPs, indicating that LCP movement models can be useful for predicting encounter locations (c.f. Balbi et al. 2019; Aiello et al. 2023; but see Iverson et al. 2024). Overall, our study indicates that it is possible to generate predictive models of road encounter locations even when data are sparse, collected unsystematically, and subject to spatial biases in reporting across a road network. Moreover, greater accuracy at locating hotspots of road encounters may be gained by combining analyses of road occurrence records with movement modeling.

We used three modeling approaches to predict road encounter locations: (1) landscape predictors identified based on all turtle road occurrence records, (2) landscape predictors identified based on hotspot clusters of records, and (3) predicted movements between wetlands based on a gene flow‐derived resistance surface. Each appears to do a fair job at identifying LULC‐associated attributes of Blanding's Turtle road encounter locations. A significant fraction of road encounter records came from a small number of hotspot sites, and these were located along or very close to the predicted LCPs; these are good candidates for immediate conservation action. For data‐poor regions, conservation agencies can treat locations that are predicted to be road encounter hotspots as priority locations for focusing survey and monitoring efforts, as possible priority candidates for mitigation.

Blanding's Turtle is a species that is inherently challenging for hotspot modeling because of its natural history. Besides having a low population density, making road encounter data scarce, Blanding's Turtle walk long distances overland (for a turtle) during the active period, both for nesting and to move between small and ephemeral wetlands. This challenge is similar for some other semiaquatic or terrestrial turtle species (e.g., Eastern Box Turtle *Terrapene carolina* , Wood Turtle *Glyptemys insculpta* ). Since at least 43% of our records appear to be associated with female nesting migrations, focusing on females during the nesting season may result in additional road encounter predictors associated with distance from potential nesting habitat such as plowed agricultural fields, grassland, and landscaped areas with bare soil. To our surprise given the differences in natural histories, predictors of Blanding's Turtle road encounter hotspots appear similar to the far more commonly encountered Painted Turtle and Common Snapping Turtle (Langen et al. 2012); it would be worthwhile to evaluate whether these are suitable surrogates to Blanding's Turtle for road encounter data.

There are growing numbers of geo‐referenced roadkill and other animal road encounter data generated by structured data collection programs (Heigl et al. 2017; Shilling et al. 2020), or else submissions of chance encounters by members of the public using popular community science applications like iNaturalist (https://www.inaturalist.org/). Such data cannot be used uncritically to make inferences about where animals encounter roads; there are severe biases in terms of survey effort across the geographic extent of the data, owing to uneven distribution of people knowledgeable and motivated to submit encounter records. This bias is likely to be most severe for smaller, infrequently encountered species that are unfamiliar to the general public. Our approach, to match encounter records with points on the same roadway to serve as comparison or pseudoabsence points, appears to be a valid way to identify what is distinctive about locations where an animal encounters a road in contrast to elsewhere along the same roadway, and presumably accessible to the animal, where there are no such records. We are sanguine that approaches similar to ours can make it possible to extract useful information about road encounter hotspots from the animal encounter databases.

Our approach of characterizing LULC around road encounter locations or location clusters and contrasting these with sites nearby along the same roadway has the potential to be misleading when roads have severe or asymmetric effects—for example, some locations that would be encounter hotspots are no longer because of local depletion (e.g., excessive roadkill causing local extirpation) or because of road avoidance (e.g., high traffic disturbance deters road approach). We assume that there are locations in the landscape where depletion has not occurred, and where traffic volumes and road configuration are not such that animals are deterred from the roadway. Should this be the case, our method will identify distinctive features associated with road encounters, and when using these to look for other sites in the full road network, may indicate places that would otherwise be hotspots but for local population depletion or deterrence because of traffic disturbance.

### 4.1 Moving Forward

Road managers and conservation agencies face a daunting problem: How to allocate scarce resources for mitigation measures on thousands of kilometers of roads in a manner that will maximize the conservation impact. Predictive road encounter models, if reasonably accurate and precise, can identify high priority road segments. These road segments may be further winnowed down by incorporating other considerations (road traffic volume, proximity to known populations of the species, land protection status in the surrounding area). From that set, managers can select the road segments most likely to be hotspots of road encounters and most impactful sites for mitigation.

We employed two modeling approaches to characterize the landscape around Blanding's Turtle road encounters. The first approach, comparing LULC and other features at road encounter locations versus matched pseudoabsence locations, is the easiest and most straightforward way to develop a predictive model. We concluded it is possible to create a reasonably predictive model even with relatively few, geographically dispersed data—typically the case with small and cryptic species in most roadkill or biodiversity databases. The second approach focused on identifying the landscape features most strongly associated with hotspots. Long‐term monitoring efforts, like the one associated with our Blanding's Turtle road encounter dataset, are often necessary to accumulate enough data within a region to identify clusters of encounters that indicate hotspots.

The density of road encounter records relative to road network length and the degree that detection biases affect the spatial pattern of records will determine what is best for analyzing the data. If there are a large number of records relative to road network length and if detection biases are minor, we recommend analyzing spatial pattern detection using KDE+ or similar methods. When data are sparse and detection biases are likely high, a paired point approach is recommended. In either case, it is possible to identify road and landscape features associated with road‐occurrence locations, and use predictive models based on those to locate places to survey as candidates for mitigation. Once constructed, the models should be applicable for locating potential hotspots on other road networks that lack road encounter data, and moreover to identify areas where depletion has occurred or road deterrence is likely impeding habitat connectivity.

Movement models that predict road encounter locations using an underlying resistance surface present an opportunity to complement and validate road encounter‐based models. Importantly, the ecological data informing the resistance surface (e.g., genetics; habitat suitability) must operate at a spatial scale that is relevant to road encounters for the focal species (Wade et al. 2015). While hotspot modeling identifies sections of a road network where animal movement is concentrated, movement models can show road locations where individuals are deterred from crossing due to factors such as traffic volume (Thurfjell et al. 2015) or behavioral avoidance (Paterson et al. 2019). In these situations, the lack of road encounter data will obscure the harmful barrier effects of the road. Though movement modeling has been used successfully for large animals (e.g., Aiello et al. 2023), whether movement models are accurate and precise enough to be useful for small animals such as turtles remains uncertain (LaPoint et al. 2013; Boyle et al. 2017). Our results indicate that they can be, based on the proximity of LCPs to road encounter hotspots and other location records. Movement models will be most effective, and may be more effective than road occurrence models, when animals have strong habitat preferences while moving through a landscape, the natural history is well‐enough characterized that the habitat preferences are known, and LULC or other habitat‐associated geographic data are available at a resolution relevant to animal movement choices. However, even if this is believed to be the case, it is important to first validate a movement model with road occurrence data.

We make the following general recommendations for using our approach to locating critical road segments for mitigation:

1. Focus analysis on areas where the species is likely to occur. Region‐focused species distribution models (e.g., Stryszowska et al. 2016) are a quantitative solution for restricting the search for road encounter hotspots to where a species is most likely to occur.
2. Apply a predictive model to identify those road segments that are most likely to be road encounter hotspots (Patrick et al. 2012). We advocate combining multiple modeling techniques to leverage the strengths of different methods, as demonstrated in this study. Targeting areas of concurrence between encounter and movement models is a more comprehensive means of locating promising sites for mitigation than relying on a single method.
3. Prune predicted hotspots in accordance with the management objectives to produce a candidate list of mitigation sites that balances cost‐effectiveness with conservation gains. Considerations might include long‐term security of the nearby populations and number of animals at risk of encountering the roadway, roadway traffic volume, long‐term stability of LULC around the hotspot, and commitment of local stakeholders to maintaining any mitigation measures.
4. Monitor selected mitigation sites before implementation of mitigation to validate their suitability and provide a baseline by which to measure the effectiveness of mitigation measures (Rytwinski et al. 2015). Monitoring may involve repeated road surveys of the putative priority hotspots to detect roadkill or roadside presence, and telemetry to document animal movement patterns in relation to these hotspots. Once validated, sites can be evaluated for specific mitigation measures such as signage, fencing, and passage structures.

## Author Contributions

**Sean G. Jackson:** formal analysis (equal), investigation (equal), methodology (equal), visualization (equal), writing – original draft (supporting), writing – review and editing (supporting). **Alexandra J. Burrows:** formal analysis (equal), investigation (equal), methodology (equal), visualization (equal), writing – original draft (supporting), writing – review and editing (supporting). **Glenn Johnson:** data curation (equal), investigation (equal), writing – review and editing (supporting). **Eric M. McCluskey:** formal analysis (supporting), methodology (supporting), writing – review and editing (equal). **Tom A. Langen:** conceptualization (lead), data curation (equal), formal analysis (equal), funding acquisition (lead), investigation (lead), methodology (equal), project administration (lead), supervision (equal), writing – original draft (lead), writing – review and editing (lead).

## Funding

This work was supported by the Northeastern Association of Fish & Wildlife Agencies.

## Conflicts of Interest

The authors declare no conflicts of interest.

## Data Availability

Georeferenced Blanding's Turtle locations are sensitive data because of active poaching for the pet trade. All Blanding's Turtle records are archived with the New York State Heritage program, where they can be accessed for legitimate research purposes. Other data in this paper are available for manuscript reviewers at https://doi.org/10.5061/dryad.fj6q5747t.
